# Plasma membrane insertion of KCa2.3 (SK3) is dependent upon the SNARE proteins, syntaxin-4 and SNAP23

**DOI:** 10.1371/journal.pone.0196717

**Published:** 2018-05-16

**Authors:** Claudia A. Bertuccio, Tony T. Wang, Kirk L. Hamilton, Diego J. Rodriguez-Gil, Steven B. Condliffe, Daniel C. Devor

**Affiliations:** 1 Department of Cell Biology, School of Medicine, University of Pittsburgh, Pittsburgh, Pennsylvania, United States of America; 2 Department of Infectious Diseases and Microbiology, Graduate School of Public Health, University of Pittsburgh, Pittsburgh, Pennsylvania, United States of America; 3 Department of Physiology, School of Biomedical Sciences, University of Otago, Dunedin, New Zealand; 4 Department of Biomedical Sciences, Quillen College of Medicine, East Tennessee State University, Johnson City, Tennessee, United States of America; Cinvestav-IPN, MEXICO

## Abstract

We previously demonstrated endocytosis of KCa2.3 is caveolin-1-, dynamin II- and Rab5-dependent. KCa2.3 then enters Rab35/EPI64C- and RME-1-containing recycling endosomes and is returned to the plasma membrane (PM). Herein, we report on the mechanism by which KCa2.3 is inserted into the PM during recycling and following exit from the Golgi. We demonstrate KCa2.3 colocalizes with SNAP-23 and Syntaxin-4 in the PM of HEK and endothelial cells by confocal immunofluorescence microscopy. We further show KCa2.3 can be co-immunoprecipitated with SNAP-23 and Syntaxin-4. Overexpression of either Syntaxin-4 or SNAP-23 increased PM expression of KCa2.3, whereas shRNA-mediated knockdown of these SNARE proteins significantly decreased PM KCa2.3 expression, as assessed by cell surface biotinylation. Whole-cell patch clamp studies confirmed knockdown of SNAP-23 significantly decreased the apamin sensitive, KCa2.3 current. Using standard biotinylation/stripping methods, we demonstrate shRNA mediated knockdown of SNAP-23 inhibits recycling of KCa2.3 following endocytosis, whereas scrambled shRNA had no effect. Finally, using biotin ligase acceptor peptide (BLAP)-tagged KCa2.3, coupled with ER-resident biotin ligase (BirA), channels could be biotinylated in the ER after which we evaluated their rate of insertion into the PM following Golgi exit. We demonstrate knockdown of SNAP-23 significantly slows the rate of Golgi to PM delivery of KCa2.3. The inhibition of both recycling and PM delivery of newly synthesized KCa2.3 channels likely accounts for the decreased PM expression observed following knockdown of these SNARE proteins. In total, our results suggest insertion of KCa2.3 into the PM depends upon the SNARE proteins, Syntaxin-4 and SNAP-23.

## Introduction

KCa2.3 is a small conductance, Ca^2+^-activated K^+^ channel known to be involved in a wide array of physiological processes [1–3]. The magnitude of the physiological response to activation of KCa2.3, which is assessed by the total current flow (I), is dictated by both the likelihood that the channels are in the open and conducting state, i.e., the open probability (P_o_) of the channel and the number (N) of channels in the plasma membrane (PM) such that IαNP_o_. Numerous studies have delved in to the regulation and gating (P_o_) of KCa2.x, as well as the related family member, KCa3.1 [4–14]. In addition, significant information regarding the mechanisms by which N is determined has now emerged. Indeed, we [15–20] and others [21–23] have identified numerous motifs in the N- and C-termini of KCa family members which are required for the proper assembly and anterograde trafficking of these channels to the PM. In addition, more recent studies have begun to shed light on the retrograde transport of KCa2.3 from the PM.

Absi et al. [24] initially demonstrated KCa2.3 resides in a caveolin-rich membrane domain in endothelial cells using both immunofluorescence and co-immunoprecipitation studies, although the endocytosis of KCa2.3 from this domain was not assessed. We first demonstrated the rapid endocytosis of KCa2.3 from the PM and further showed the channel was rapidly recycled back to the PM in a Rab35/EPI64C/RME-1-dependent manner in both HEK cells and HMEC-1 endothelial cells [25]. In a subsequent study, we showed the endocytosis of KCa2.3 from the PM is dependent upon both caveolin-1 and dynamin II, consistent with caveolar localization [26]. We further demonstrated KCa2.3 was initially endocytosed in to Rab5-containing early endosomes [26]. Indeed, perturbation of these pathways led to increased PM KCa2.3 as a result of a reduced endocytic rate [26]. Further, Lin et al. [27] demonstrated that disruption of the cholesterol-rich domains in endothelia with methyl-β-cyclodextrin inhibited the endocytosis of KCa2.3 and this process was regulated by changes in intracellular Ca^2+^.

In the present study, we investigated the role of Soluble NSF Attachment protein REceptor (SNARE) proteins in the re-insertion of KCa2.3 in to the PM following endocytosis as well as in the insertion of KCa2.3 in to the PM following Golgi exit. We demonstrate Syntaxin-4 and Soluble NSF Attachment Protein (SNAP)-23 co-localize with KCa2.3 in the PM and knockdown of SNAP-23 inhibits both KCa2.3 recycling following endocytosis as well as Golgi-to-PM trafficking. Based on these studies, combined with previous reports, we propose a model for the protein complexes involved in the recycling of KCa2.3 at the PM.

## Materials and methods

### Molecular biology

The biotin ligase acceptor peptide (BLAP)-tagged KCa2.3 construct has been previously described [25]. BLAP-KCa2.3 replication deficient adenoviruses were generated by the University of Pittsburgh Vector Core facility. The BirA-KDEL adenovirus was generously provided by Dr. Alexander Sorkin, (University of Pittsburgh, Pittsburgh, PA). KCa2.3 and myc-tagged KCa2.3 were a generous gift from J.P. Adelman (Vollum Institute, Oregon Health Sciences University). GFP-tagged syntaxin-4 and GFP-tagged SNAP-23 cDNAs were obtained from OriGene. The fidelity of all constructs utilized in this study was confirmed by sequencing (ABI PRISM 377 automated sequencer, University of Pittsburgh).

### Cell culture

Human embryonic kidney (HEK293) and HeLa cells were obtained from the American Type Culture Collection (Manassas, VA) and cultured in Dulbecco’s modified Eagle’s medium (DMEM; Invitrogen, Carlsbad, CA) supplemented with 10% fetal bovine serum and 1% penicillin-streptomycin (10,000 Units/ml) in a humidified 5% CO_2_/95% O_2_ incubator at 37°C. Cells were transfected using LipofectAMINE 2000 (Invitrogen) following the manufacturer’s instructions. In some experiments, after transfection with the scrambled shRNA, Syntaxin-4 shRNA or SNAP-23 shRNA, HEK293 cells were transduced with BirA-KDEL and BLAP-KCa2.3 adenoviruses. Briefly, HEK293 cells were washed twice with DMEM growth media followed by the addition of adenoviruses. After 1h of incubation at 37°C in a 5% CO_2_/95% O_2_ atmosphere, cells were washed twice with DMEM growth media and allowed to recover until the next day.

The immortalized hCMEC/D3 endothelial cell line was kindly provided by Dr. Weksler (Cornell University). The culture ware was coated with rat tail collagen type I (R&D systems) at a concentration of 150 ug/ml for 1 hour at 37°C according to the manufactures instructions. Endothelial cells were grown in EBM-2 basal medium (Lonza Bioscience) supplemented with EGM-2 containing bFGF (Sigma), ascorbic acid (Sigma), hydrocortisone (Sigma), HEPES and Chemically Defined Lipid Concentrate (Invitrogen) and incubated at 37°C with 5% CO_2_/95% O_2_. hCMEC/D3 cells were transduced with BLAP-KCa2.3 adenovirus, as above, as indicated for each experiment.

### Immunoprecipitations (IP), immunoblots (IB), biotinylation of KCa2.3 using BirA and total KCa2.3 plasma membrane expression

Our IP and IB protocols have been described, in detail [25,26,28–31]. Biotin ligase (BirA) was expressed from pET21a-BirA (generously provided by Dr. Alice Y. Ting, Massachusetts Institute of Technology, Cambridge, MA) in *E*. *coli*, as described [25]. Cell surface, BLAP-tagged KCa2.3 was labeled with biotin and subsequently streptavidin, as previously described, in detail [25,26,28–30]. The cells were then lysed and equivalent amounts of lysate were immunoprecipitated with α-streptavidin antibody followed by IB for SNAP-23 or Syntaxin-4, as above. Evaluation of total PM KCa2.3 expression was carried out via cell-surface biotinylation, using EZ-Link Sulfo-NHS-SS-Biotin (Thermo Scientific, Rockford, IL), as previously described [25,26]. Relative protein levels were quantified by densitometry using ImageJ software (NIH; http://rsb.info.nih.gov/ij/).

### Antibodies

α-KCa2.3 Ab was obtained from Chemicon (Temecula, CA), α-myc Ab (9E10) was obtained from Covance (Richmond, CA), α-GFP Ab was obtained from Santa Cruz Biotechnology (Santa Cruz, CA), monoclonal α-streptavidin and α-SNAP-23 (AB4114) antibodies were obtained from Abcam (Cambridge, MA) and mouse α-syntaxin-4 was obtained from BD Transduction laboratories (San Jose, CA).

### Short-hairpin RNA treatment (shRNA)

Putative shRNA sequences were designed against SNAP-23 and Syntaxin-4 mouse genes using web software provided by Dharmacon RNA Technologies (http://www.dharmacon.com/). Sequences were determined to be unique to the gene by BLAST searches of the GenBank database. Oligonucleotide sequences were designed corresponding to the sense and antisense sequences of the shRNA target sites of interest separated by a hairpin loop sequence (SNAP-23 sense: 5’-GGGCAGTATCCTGGGAAATCTTCCTGTCAATTTCCCAGGATACTGCCC-3’, Syntaxin-4 sense: 5’-GGAGGAAGCTGATGAGAACTATACTTCCTGTCATATAGTTCTCATCAGCTTCCTCC-3’). The oligonucleotide pairs were then annealed and cloned into the linearized pGeneClip^™^ hMGFP Vector using the GeneClip^™^ U1 Hairpin Cloning Systems (Promega), according to the manufacturer’s instructions. Scrambled shRNA was used as control. HeLa cells were plated at ~40% confluence and transfected twice over a 24hr period with scrambled, SNAP-23 or Syntaxin-4 shRNAs using LipofectAMINE 2000, according to the manufacturer’s directions. The third day, the cells were transduced with BirA-KDEL and/or BLAP-KCa2.3 adenoviruses, as described.

### Recycling assay

Our KCa2.3 recycling assay was carried out as previously described, in detail [25]. These experiments utilized HeLa cells that had been transfected with scrambled or SNAP-23 shRNAs, followed by BLAP-KCa2.3 adenovirus transduction. Following cell surface biotinylation, as above, the cells were warmed to 37°C to allow channel endocytosis for 30 min (T = 0). The remaining cell surface biotin was stripped using MESNA (100 mM sodium 2-mercaptoethanesulfonate, 50 mM Tris, 100 mM NaCl, 1 mM EDTA, 0.2% BSA) 3 times per 10 min. each at 4°C after which the cells were lysed and the protected (endocytosed) biotin-tagged channels subjected to pulldown using streptavidin-agarose following normalization of protein concentrations. The endocytosed samples were compared to samples which were either biotinylated and immediately subjected to lysis and streptavidin pulldown (control) or biotinylated and immediately stripped to confirm the efficiency of the MESNA stripping step (strip). Subsequent to endocytosis, the cells were rewarmed to 37°C for either 10 min or 30 min to allow endocytosed KCa2.3 to recycle. The channels which have returned to the cell surface were then stripped of biotin using MESNA, as above, and the remaining non-recycling channels quantified by pull down and IB, as above. In this case, a decrease in signal over time is indicative of recycling, as previously described [25].

### KCa2.3 trafficking from Golgi to the plasma membrane

These studies were carried out as described for KCa3.1 [28]. Briefly, HeLa cells were transfected with scrambled or SNAP-23 shRNAs (see above) and subsequently transduced with the BLAP-KCa2.3 adenovirus. In these experiments, BLAP-KCa2.3 was biotinylated in the ER by co-transduction of BirA-KDEL. Plasma membrane BLAP-KCa2.3 was then labeled with non-conjugated streptavidin (Invitrogen) for 15 min at 4°C, followed by an extensive wash to remove unbound streptavidin and then cell lysis (T = 0). In another set of dishes, neutravidin biotin binding protein (600 μg/ml) (Thermo Scientific, Rockford, IL) was added for 2 hrs at 4°C such that all plasma membrane localized BLAP-KCa3.1 channels would be bound and “blocked” from binding to subsequently added streptavidin (Block). In order to allow accumulation of channels in the Golgi, cells were incubated for 2 hrs at 19°C in the continued presence of neutravidin. Cells were then washed twice on ice-cold PBS, warm media was added and the cells were incubated for 20 min (T = 20’) or 2 hrs (T = 2 hrs) at 37°C to allow channels to traffic to the plasma membrane. Finally, the newly resident plasma membrane channels were labeled with non-conjugated streptavidin followed by IB with α-streptavidin antibody to quantify the rate of plasma membrane KCa2.3 appearance in the absence or presence of endogenous SNAP-23 and Syntaxin-4 [28].

### Preparation of PM footprints

Plasma membrane footprints of hCMEC/D3 cells were obtained using a modification of the cationic colloidal silica microbead membrane-isolation procedure [31]. Briefly, endothelial cells were washed twice with PBS and washed again in coating buffer (CB; 135 mM NaCl, 20 mM 2[N-morpholino]ethanesulfonic acid, 1 mM Mg^2+^, 0.5 mM Ca^2+^, pH 5.5). The cells were coated with a 1% (w/v) cationic colloidal silica solution in CB for 2 min. and then coated using a 1 mg/ml polyacrylic acid solution in CB at pH 5.0 for 2 min. Cells were washed twice with CB, then washed quickly in domain lysis buffer (DLB; 2.5 mM imidizole, pH 7.5, containing 1 mM Mg^2+^ and 0.5 mM Ca^2+^ and Complete^™^ EDTA-free protease inhibitor cocktail mix). Cells were subsequently incubated in DLB on ice for 30 min. Using a 5 ml syringe fitted with a shortened, flattened, blunt 18 gauge needle, shear forces were applied to the cells by squirting the cells with DLB at a 45° angle such that the top of the cell and intracellular components were sheared away. The plasma membrane that remained attached to the glass-bottom dish was washed twice with DLB. For labeling, plasma membrane domains were fixed with 2% paraformaldehyde in PBS and the remaining membranes were labeled as described below.

### Confocal microscopy

hCMEC/D3 cells were grown in a 35 mm glass-bottom dish (Mat-Tek Co) and transduced with BLAP-KCa2.3 adenovirus. Subsequent to the generation of PM footprints, the remaining membrane was enzymatically biotinylated and streptavidin-Alexa555 labeled for 15 min at 4°C. The PM footprints were extensively washed with PBS/BSA to remove unbound streptavidin. The PM footprints were subsequently blocked with 10% goat serum, 0.5% BSA in PBS for 30 min at 4°C, and incubated with anti- SNAP-23 or anti-Syntaxin-4 antibodies overnight at 4°C after which they were incubated with goat α-rabbit-Alexa488 or goat α-mouse-Alexa488, respectively for 1 hr at 4°C and subsequently imaged by laser confocal microscopy using an Olympus FluoView 1000.

Colocalization analysis between KCa2.3 and SNAP-23 or Syntaxin-4, was done by tracing a line across the cell using Image J [32]. The relative optical density along the line (in arbitrary units) for each channel was plotted. The total number of peaks for each channel individually was manually counted and after that, the number of peaks showing expression of both markers was determined. For each cell four randomly placed, non-crossing lines were analyzed, and three cells were analyzed per condition. To do the statistical analyses we considered two possible scenarios: a- these proteins never interact and therefore the expected colocalization value should be close to 0% or, b- these proteins diffuse in the plasma membrane independently of each other and randomly they are close enough to exhibit colocalization, in which the expected value would be ~50%. Therefore, t-tests comparing our colocalization data vs 0% or 50% were done separately for each one of the possible scenarios.

### Patch-clamp electrophysiology

HeLa cells were plated onto 10 mm glass coverslips pre-treated with poly-L-lysine. After 24 hrs, cells were transfected with KCa2.3 and GFP (control) or SNAP-23 or a scrambled control shRNAs, as described above. Between 2–4 days after transfection, whole-cell K^+^ currents were recorded from GFP positive cells in an external solution containing (in mM): 130 NaCl, 4 KCl, 2 CaCl_2_, 1 MgCl_2_, 10 glucose and 10 HEPES, pH 7.4 with NaOH. Patch pipettes (2–3 MΩ resistance) were filled with internal solution (in mM): 100 K-gluconate, 20 KCl, 8.75 CaCl_2_, 1 MgCl_2_, 10 EGTA, 10 HEPES, 4 Mg-ATP and 0.3 Tris-GTP that had a calculated free Ca^2+^ concentration of 1μM. These were connected to an Axon Axopatch 200B amplifier (Molecular Devices) interfaced to a PC via a Digidata 1320 (Molecular Devices). Upon achieving the whole cell configuration, cells were voltage-clamped at -80mV and control K^+^ currents were generated by a 500 ms voltage ramp from -100 to 60 mV using pClamp10 software (Molecular Devices) where data were acquired at 5 kHz. Cells were then perfused with external solution containing 300 nM apamin and apamin-sensitive currents were calculated offline by subtracting the current remaining post-apamin from the control K^+^ current at 0 mV. Series resistance was routinely compensated at 60–75% and access resistance was continually monitored during the experiment where recordings with significant deviations were excluded from analysis.

### Chemicals

All chemicals were obtained from Sigma-Aldrich, unless otherwise stated.

### Statistics

All data are presented as means ± SEM, where *n* indicates the number of experiments. Statistical analysis was performed using a Student’s t test. To compare the normalized values of the IB band intensities, statistical analysis was performed using the non-parametric Kruskal-Wallis test. A value of P<0.05 is considered statistically significant and is reported.

## Results

**Fig 1:**
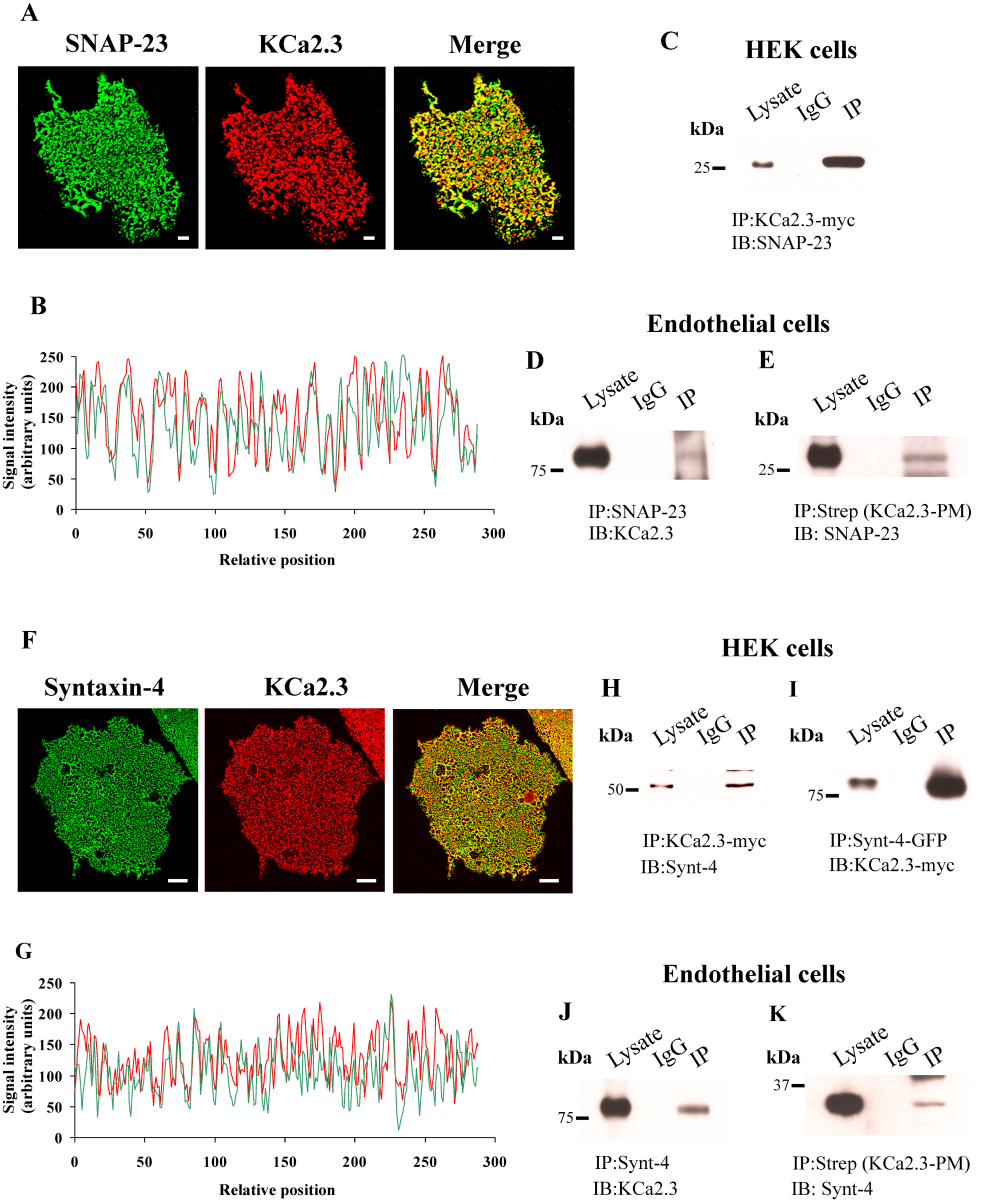
**(A)** Co-localization of KCa2.3 with SNAP23 in hCMEC/D3 endothelial cells membrane footprints. hCMEC/D3 cells were transduced with BLAP-KCa2.3 Adenovirus. KCa2.3 was labeled with streptavidin-Alexa555 (Red, middle panel) and endogenous SNAP23 was labeled with α-SNAP23 Ab (Green, left panel). Co-localization is shown in yellow (Merge, Right panel). **(B)** Tracing analysis showing KCa2.3 (red) exhibits colocalization with SNAP-23 (green). **(C)** HEK cells were transfected with myc-tagged KCa2.3. KCa2.3 was immunoprecipitated followed by blotting for endogenous SNAP23. **(D** and **E)** hCMEC/D3 cells were transduced with BLAP-KCa2.3 Adenovirus. In **(D)** endogenous SNAP23 was immunoprecipitated followed by blotting for KCa2.3. In **(E)**, plasma membrane (PM) localized KCa2.3 was immunoprecipitated using α-streptavidin Ab followed by blotting for endogenous SNAP23. **(F)** Co-localization of KCa2.3 with Syntaxin-4 (Syn4) in hCMEC/D3 endothelial cell membrane footprints, as above. KCa2.3 was labeled with streptavidin-Alexa555 (Red, middle panel) and endogenous Syn4 was labeled with α-Syn4 Ab (Green, left panel). Co-localization is shown in yellow (Merge, Right panel). **(G)** Tracing analysis showing KCa2.3 (red) exhibits colocalization with Syn4 (green). **(H** and **I)** HEK cells were transfected with myc-tagged KCa2.3 and GFP-tagged Syn4. In **(H)**, KCa2.3 was immunoprecipitated followed by blotting for Syn4. In **(I)**, Syn4 was immunoprecipitated followed by blotting for KCa2.3. **(J** and **K)** hCMEC/D3 cells were transduced with BLAP-KCa2.3 Adenovirus. In **(J)**, endogenous Syn4 was immunoprecipitated followed by blotting for KCa2.3. In **(K)**, plasma membrane (PM) localized KCa2.3 was immunoprecipitated using α-streptavidin Ab followed by blotting for endogenous Syn4. In all blots, the lysate is shown in lane 1, the IgG control is shown in lane 2 and the IP is shown in lane 3. Scale bar = 10 μm in A and F.

It has previously been demonstrated that KCa2.3 is localized to caveolin-rich domains in the PM [24,26,27]. Similarly, in endothelial cells, the PM SNARE proteins, syntaxin-4 and SNAP-23, have been shown to reside in these caveolin-rich membrane domains [33]. Thus, we initially determined whether KCa2.3 co-localizes with syntaxin-4 and SNAP-23 in the PM of endothelial cells. These studies were carried out using heterologously expressed BLAP-tagged KCa2.3 and endogenously expressed syntaxin-4 or SNAP-23 on membrane footprints, as this allowed us to unequivocally evaluate co-localization solely in the PM. As shown in Fig 1, both SNAP-23 (A) and syntaxin-4 (F) displayed a punctate labeling pattern, as previously described [33]. We then analyzed signal intensity of the red (KCa3.2) and green (SNAP-23 or Synt-4) markers in the PM of endothelial cells by tracing arbitrary lines, and determined the relative optical density (fluorescence) for each of the antibodies. As shown in Fig 1B and 1G, the tracing of KCa3.2 exhibits peaks of expression that align with the peaks of expression shown on the tracing of SNAP-23 or Synt-4. KCa3.2 showed a significant level of colocalization with SNAP-23 of 82±1% when analyzed versus hypothetical no interaction (P<0.0001 vs 0%) or random interaction (P<0.0001 vs 50%). KCa3.2 showed a significant level of colocalization with Synt-4 of 79±1% when analyzed versus hypothetical no interaction (P<0.0001 vs 0%) or random interaction (P<0.0001 vs 50%). These experiments demonstrate that KCa2.3 displayed co-localization with both SNAP-23 and syntaxin-4, consistent with these proteins being expressed together in caveolin-rich domains.

Based on these co-localization studies, we determined whether KCa2.3 could be co-immunoprecipitated with these SNARE proteins in both HEK and endothelial cells. As shown, we were able to detect SNAP-23 in the myc-KCa2.3 IP from HEK cells (Fig 1C) and we similarly were able to IP SNAP-23 from endothelial cells and detect KCa2.3 by IB (Fig 1D). While these experiments demonstrate we can show a close association between KCa2.3 and SNAP-23 in total lysate, we also wanted to determine whether these proteins were closely associated in the PM, as suggested by our co-localization studies. Thus, we specifically immunoprecipitated PM localized BLAP-KCa2.3, following specific biotinylation with BirA and immunoprecipitation with streptavidin antibody [25,26], and subsequently blotted for SNAP-23. As shown in Fig 1E, PM KCa2.3 co-IPs with SNAP-23, consistent with these proteins existing in the same complex in the PM. Similar experiments were carried out with syntaxin-4 and KCa2.3 in HEK and endothelial cells. In HEK cells we co-expressed myc-KCa2.3 and GFP-syntaxin-4 and either immunoprecipitated KCa2.3 (Fig 1H) or syntaxin-4 (Fig 1I) and blotted for syntaxin-4 or KCa2.3, respectively. As is apparent, in both instances, we were able to demonstrate an association between KCa2.3 and syntaxin-4. In endothelial cells we also demonstrated a co-IP between endogenous syntaxin-4 and KCa2.3 following IP of syntaxin-4 (Fig 1J). As with SNAP-23, we confirmed that PM localized KCa2.3 could be immunoprecipitated with syntaxin-4 (Fig 1K), confirming these proteins associate in the PM.

**Fig 2:**
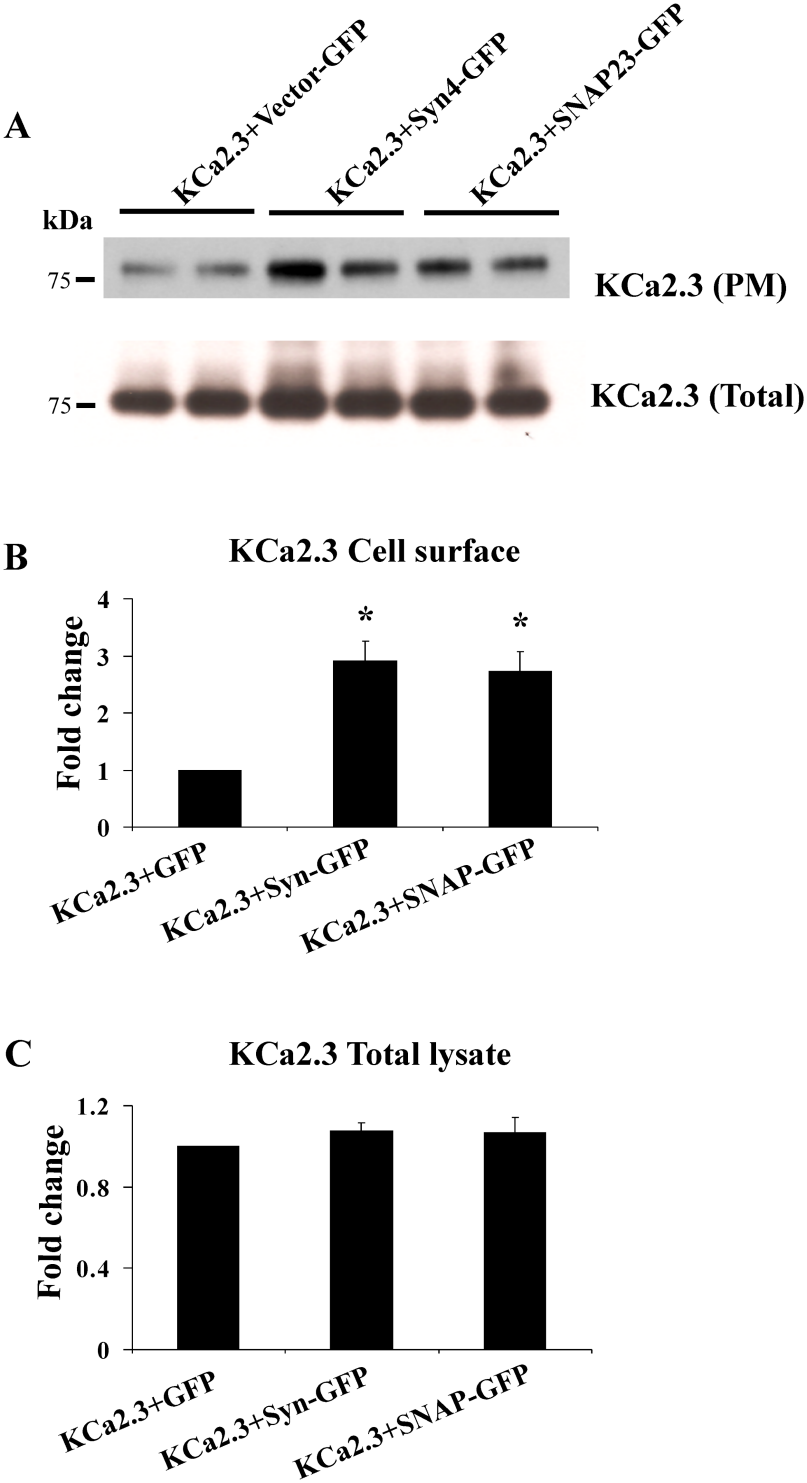
Overexpression of syntaxin-4 (Syn4) or SNAP-23 increase plasma membrane (PM) expression of KCa2.3 in HEK cells. **(A)** HEK cells were transfected with BLAP-KCa2.3 and GFP-tagged Syn4 or SNAP-23. PM KCa2.3 (top blot) was assessed by specifically biotinylating PM localized KCa2.3 and streptavidin labeling, followed by cell lysis and blotting for streptavidin (see Methods). Total KCa2.3 expression (bottom blot) was determined by blotting with α-KCa2.3 Ab. Lysates from 2 separate experiments were run together in the same blot. As is apparent, both Syn4 and SNAP-23 overexpression increased PM KCa2.3 expression while having no effect on total KCa2.3 expression. Densitometries were determined and plotted for a total of 6 separate experiments in **(B)** (plasma membrane KCa2.3) and **C** (total KCa2.3).

Given the role of these SNARE proteins in vesicle fusion, we anticipated modifying their expression would alter PM expression of KCa2.3. Initially, we overexpressed either syntaxin-4 or SNAP-23 together with BLAP-KCa2.3 in HEK cells and determined PM KCa2.3 expression levels. As shown in Fig 2, overexpression of either syntaxin-4 or SNAP-23 significantly increased expression of PM localized KCa2.3 (A, B) while having no effect on the total expression of KCa2.3 (A, C). In 3 experiments, syntaxin-4 overexpression increased PM KCa2.3 expression an average of 2.9±0.3-fold (P<0.05), whereas overexpression of SNAP-23 increased KCa2.3 expression 2.7±0.3-fold (P<0.05).

**Fig 3:**
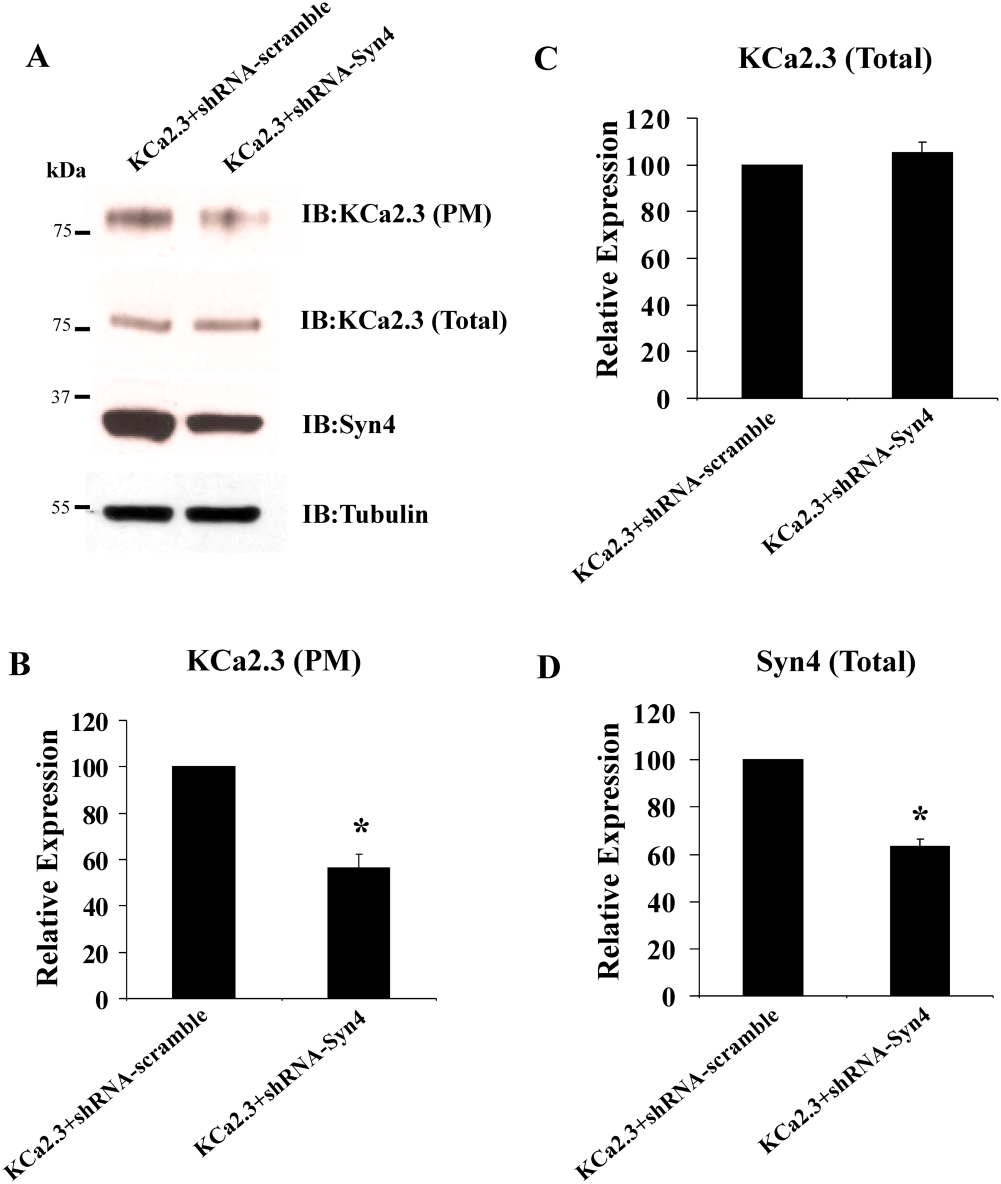
shRNA-mediated knockdown of syntaxin-4 (Syn4) decreases expression of KCa2.3 at the plasma membrane (PM) in HeLa cells. **(A)** HeLa cells were transfected with either scrambled shRNA (left lane) or shRNA directed against Syn4 (right lane), followed by transfection with KCa2.3 (see Methods). Knockdown of Syn4 was confirmed by immunoblot (bottom blot and panel **(D)**). Tubulin was used as a loading control. Knockdown of Syn4 resulted in a decreased PM expression of KCa2.3 (top blot and panel **(B)**), whereas total KCa2.3 expression was unaffected (middle blot and panel **(C)**). Experiments were repeated 3 times, densitometries determined and plotted in panels **(B-D)**.

**Fig 4:**
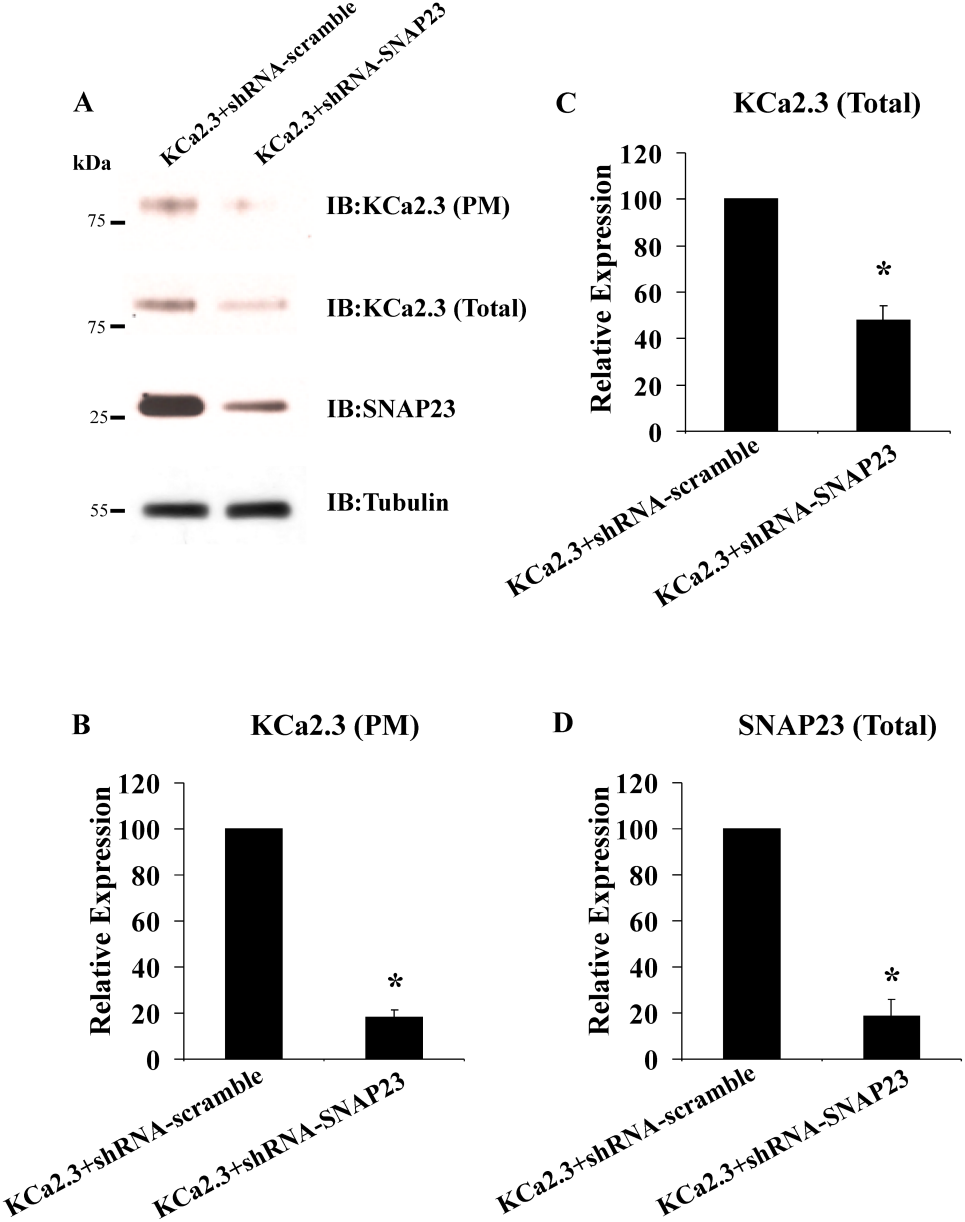
shRNA-mediated knockdown of SNAP23 decreases expression of KCa2.3 in HeLa cells. **(A)** HeLa cells were transfected with either scrambled shRNA (left lane) or shRNA directed against SNAP23 (right lane), followed by transfection with KCa2.3 (see Methods). Knockdown of SNAP23 was confirmed by immunoblot (bottom blot and panel **(D)**). Tubulin was used as a loading control. Knockdown of SNAP23 resulted in a decrease in both PM expression of KCa2.3 (top blot and panel **(B)**), as well as total KCa2.3 (middle blot and panel **(C)**). Experiments were repeated 3 times, densitometries determined and plotted in panels **(B-D)**.

Our initial attempts to knock down syntaxin-4 and SNAP-23 protein expression using either siRNAs or shRNAs in HEK cells failed to produce a significant decrease in expression of these proteins. However, others reported successful knockdown of syntaxin-4 and SNAP-23 in HeLa cells [34] and thus we evaluated whether we could successfully suppress syntaxin-4 and SNAP-23 protein expression in these cells. As shown in Figs 3(A), 3(D), 4(A) and 4(D), shRNAs directed against syntaxin-4 and SNAP-23, respectively resulted in a significant reduction in SNARE expression, relative to scrambled shRNA. In 3 experiments, we achieved a 36±3% reduction in syntaxin-4 (Fig 3D) and an 81±7% reduction in SNAP-23 (Fig 4D; P<0.05). As shown in Fig 3(A)–3(C), syntaxin-4 knockdown resulted in a decrease in PM KCa2.3 expression of 56±6%, while having no effect on total KCa2.3 expression (105±5%). Similarly, SNAP-23 knockdown resulted in a significant decrease in PM KCa2.3 (Fig 4A and 4B), averaging 82±3% (P<0.05; Fig 4B). However, SNAP-23 knockdown also resulted in a decrease in total KCa2.3 expression, averaging 52±6% (P<0.05; Fig 4C). These results clearly demonstrate that knockdown of either syntaxin-4 or SNAP-23 results in a significant decrease in KCa2.3 expression at the PM.

**Fig 5:**
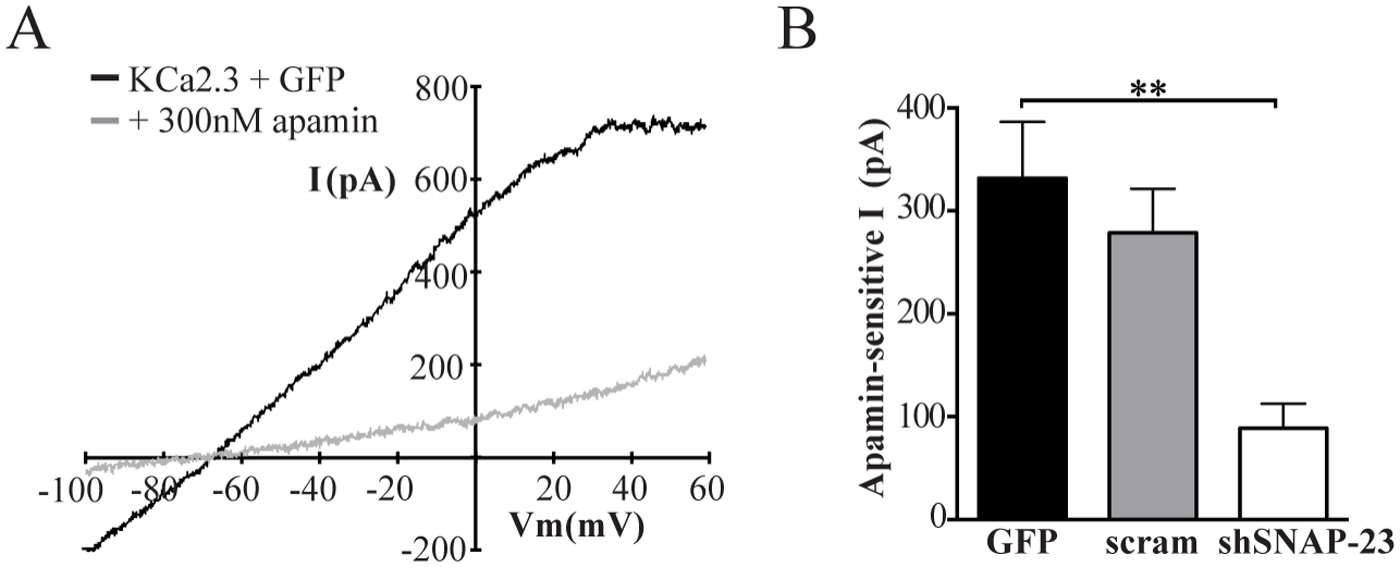
Knockdown of SNAP-23 reduces whole-cell KCa2.3 current in HeLa cells. **(A)** Representative current traces (top) in response to a voltage ramp (below) in HeLa cells expressing KCa2.3 and GFP before (solid line) and after (dashed line) exposure to 300 nM apamin. **(B)** Mean apamin-sensitive current in HeLa cells transfected with KCa2.3 and GFP or SNAP-23 shRNA or scrambled shRNA measured at 0 mV (*n* = 7–10, where * indicates *p* < 0.05).

As SNAP-23 knockdown resulted in the larger change in KCa2.3 expression, we carried out the remainder of our studies following SNAP-23 knockdown in HeLa cells. To assess whether the decrease in PM KCa2.3 observed with our biochemical assays correlated with a change in functional KCa2.3 expression we carried out whole-cell patch-clamp studies following expression of KCa2.3 with either GFP, scrambled shRNA or shRNA directed against SNAP-23. Fig 5A shows representative recordings from cells expressing KCa2.3 and GFP before and after exposure to the specific KCa2.3 blocker, apamin. A large, rectified current is generated in the KCa2.3 plus GFP cell in response to the indicated voltage ramp protocol that reversed at -80 mV, near the Nernst potential for K^+^ under these conditions. Perfusion of 300 nM apamin caused a rapid decrease in baseline K^+^ currents (Fig 5A) indicating it was specifically conducted by exogenous KCa2.3 (non-transfected cells had minimal apamin sensitive current; data not shown). Analysis of the mean data demonstrates transfection of KCa2.3 with shRNA against SNAP-23 significantly reduced the amount of apamin-sensitive current compared to KCa2.3 plus GFP controls (Fig 5B). In contrast, a scrambled shRNA sequence had no effect (Fig 5B). These results confirm there is a decrease in amount of functional KCa2.3 channels at the plasma membrane when SNAP-23 expression is reduced.

**Fig 6:**
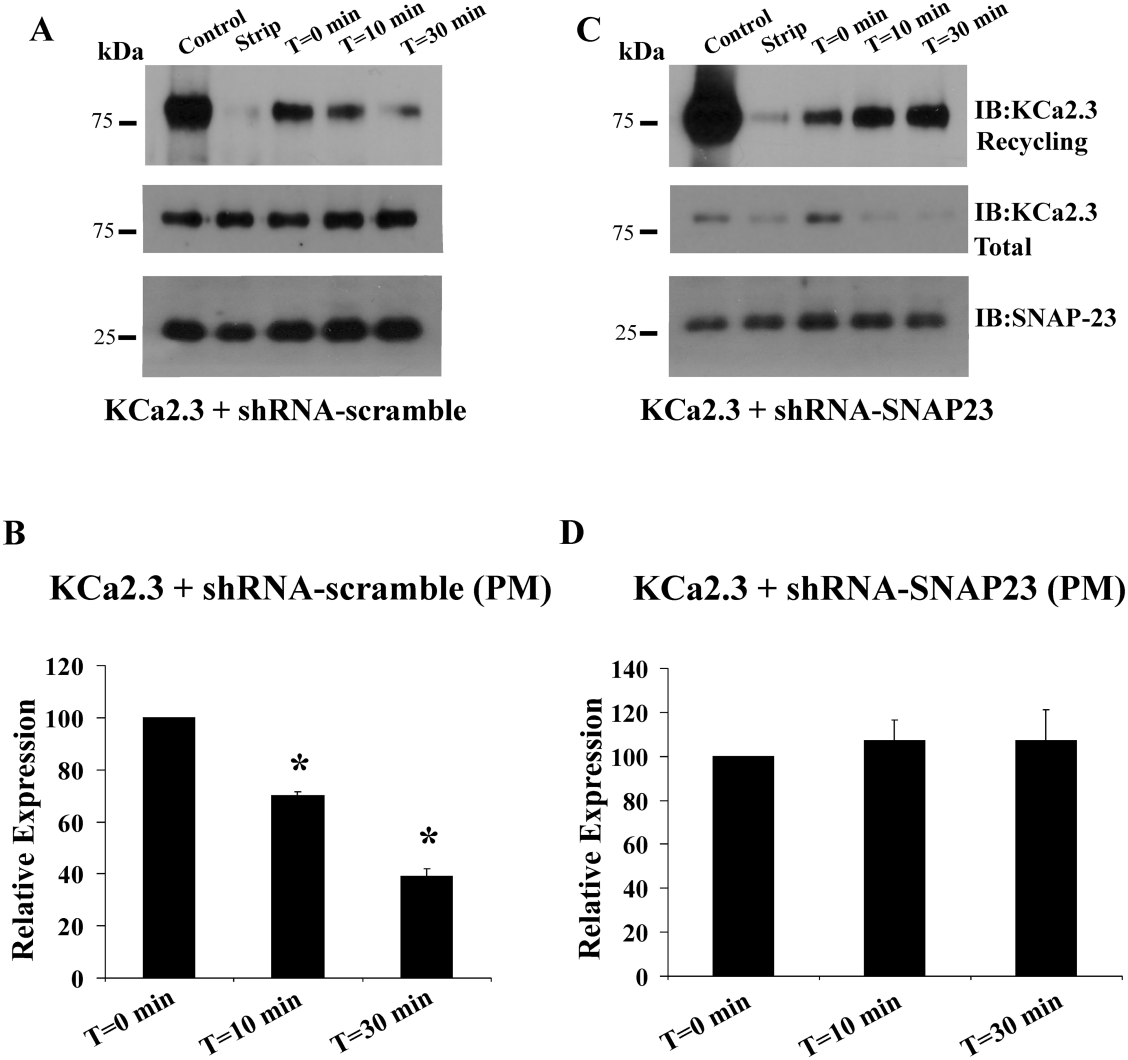
shRNA-mediated knockdown of SNAP23 blocks recycling of KCa2.3 to the plasma membrane (PM) in HeLa cells. HeLa cells were transfected with KCa2.3 and PM recycling assessed as detailed in the Methods. **(A)** In the presence of a scrambled shRNA 61% of KCa2.3 recycled back to the PM after 30 min, as assessed by the loss of KCa2.3 signal. **(B)** Densitometries for 3 experiments using scrambled shRNA are plotted. **(C)** Following shRNA-mediated knockdown of SNAP23 (compare bottom blots in **(A and C)**) KCa2.3 continued to be endocytosed, but failed to recycle back to the PM as assessed by the consistent KCa2.3 band intensities at 0 and 30 min (top blot). Note that total KCa2.3 was reduced following knockdown of SNAP23 (middle blot). **(D)** Densitometries for 3 experiments using shRNA directed against SNAP23 are plotted.

The decrease in PM KCa2.3 expression observed both biochemically and electrophysiologically following knockdown of SNAP-23 could be caused by either an inhibition of channel recycling or an inhibition of Golgi-to-PM trafficking. A representative experiment for the recycling of KCa2.3 is shown in Fig 6A. Following cell surface biotinylation, the cells were separated in to three groups. One plate of cells was immediately lysed and subject to streptavidin pulldown followed by SDS-PAGE and IB for KCa2.3 (control, lane 1). A second plate of cells was immediately subject to the biotin stripping protocol to evaluate the efficiency of this step (strip, lane 2). The remaining plates of cells were returned to 37 °C for a 30 min incubation to allow the biotinylated, PM KCa2.3 to endocytose to steady-state [25] after which the remaining biotinylated channel at the PM was stripped at 4 °C. One plate of cells was then lysed and subject to streptavidin pulldown followed by KCa2.3 IB to assess the amount of channel endocytosed during this period: this is referred to as T = 0 for these studies. Subsequently, the remaining plates of cells were returned to 37 °C for either 10 or 30 min to allow the endocytosed channel to have the opportunity to recycle back to the PM, after which these channels, which would still be biotinylated, are once again subject to stripping of the bound biotin. In this way, a decrease in signal over time is reflective of channel recycling [25]. As shown in Fig 6 (bottom blot), we achieved significant knockdown of SNAP-23 in these studies, compared to scrambled shRNA (Fig 6A, bottom blot) as well as a decrease in total KCa2.3 (compare middle blots), consistent with our results above. Note that we cannot compare our initial PM expression levels (control) for these experiments as we pulled down from more total protein to increase our signal for these recycling studies given the lower PM expression observed following knockdown of SNAP-23 (Fig 4). Importantly, in the presence of the scrambled shRNA, KCa2.3 recycled back to the PM. Indeed, after 30 min, 61±4% (n = 3, P<0.05) of the channel had recycled (Fig 6A, top panel and Fig 6B), similar to what we previously described [25]. In contrast, following knockdown of SNAP-23 (Fig 6C, top panel and Fig 6D), KCa2.3 completely failed to recycle (107±14%, n = 3). These results demonstrate that SNAP-23 is critical to the recycling of KCa2.3.

**Fig 7:**
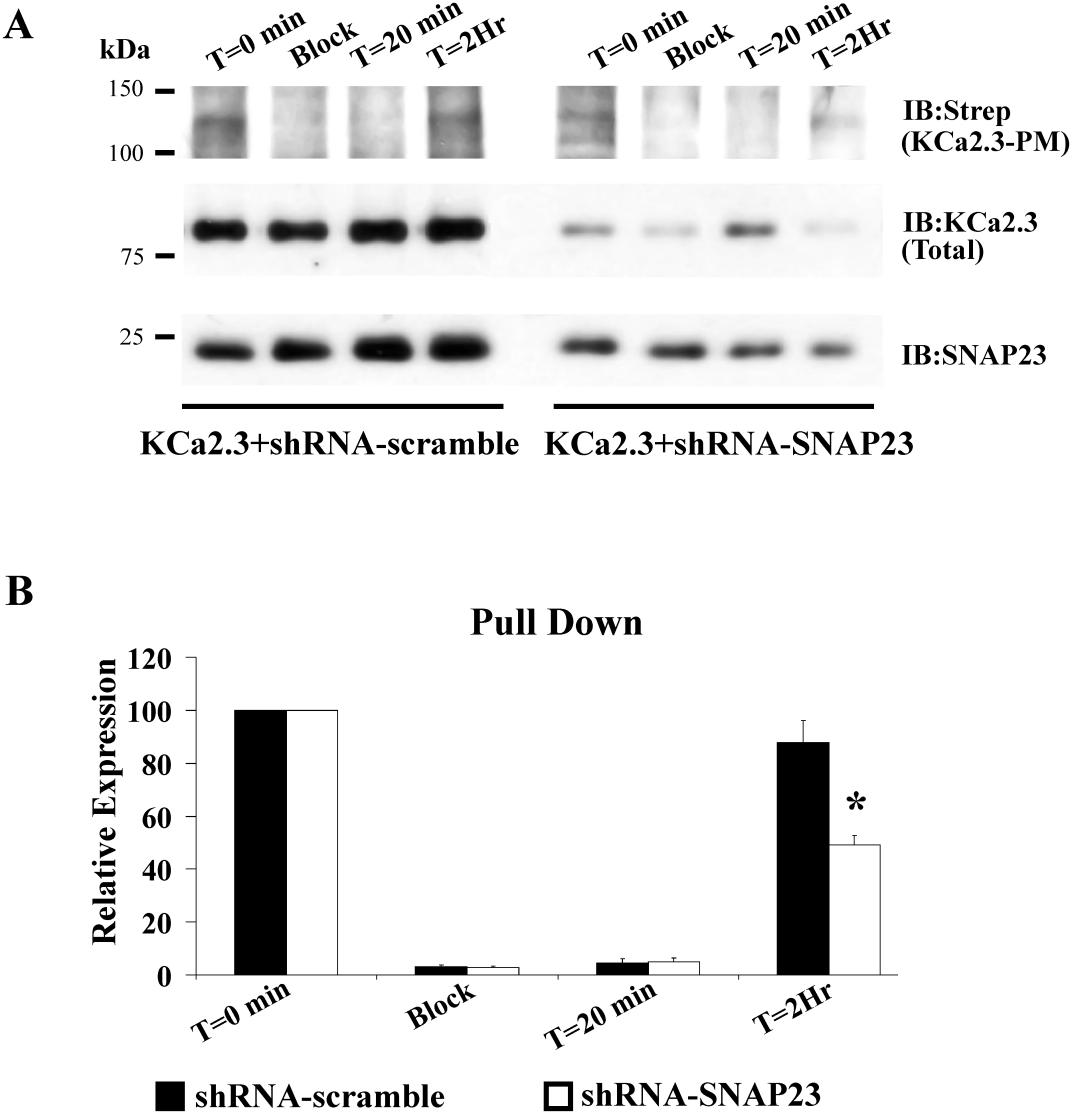
shRNA-mediated knockdown of SNAP23 slows Golgi to plasma membrane (PM) delivery of KCa2.3 in HeLa cells. **(A)** HeLa cells were transfected with BLAP-KCa2.3/BirA-KDEL such that KCa2.3 channels are biotinylated in the ER. Neutravidin was used to “block” the KCa2.3 channels at the PM after which newly arrived KCa2.3 channels could be “captured” with streptavidin (see Methods). Following block with neutravidin, no channels were detected at the PM. Subsequent incubation at 37 °C for 2 hrs resulted in new channels being trafficked from the Golgi to the PM. Following shRNA-mediated knockdown of SNAP23 less KCa2.3 trafficked to the PM after 2 hrs compared to the scrambled shRNA control. Blots of total KCa2.3 confirmed decreased expression following SNAP23 knockdown (middle blot). SNAP23 knockdown was confirmed by immunoblot (bottom blot). **(B)** Average densitometries for 3 experiments as shown in A. Solid bars are scrambled shRNA and open bars are SNAP23 shRNA.

The forward trafficking of KCa2.3 was carried identically to what we previously described for KCa3.1 [28] and described in the Methods. For these studies, we co-expressed BLAP-KCa2.3 with BirA-KDEL plus either scrambled shRNA or shRNA directed against SNAP-23. In this way, KCa2.3 will be specifically biotinylated in the ER. The results of these studies are shown in Fig 7. Steady-state PM KCa2.3 was determined by streptavidin labeling and subsequent IB (T = 0), as described [28]. In an additional plate of cells, PM localized KCa2.3 was “blocked” with neutravidin after which streptavidin was added to “capture” any unblocked channels resident in the membrane to assess the efficiency of this blocking step (Block). As is apparent, we were unable to detect KCa2.3 under these blocked conditions, indicating neutravidin efficiently binds and inhibits streptavidin binding to the biotinylated channel. Additional plates of neutravidin blocked cells were subsequently incubated at 19°C, in the continued presence of neutravidin, such that biotinylated KCa2.3 would accumulate in the Golgi. An additional incubation at 37°C, in the absence of neutravidin, for either 20 min or 2 hrs allowed KCa2.3 to traffic to the PM. These newly arrived channels were then “captured” using streptavidin and quantified by IB. As shown in Fig 7, in 3 experiments in the presence of scrambled shRNA, KCa2.3 accumulated at the PM similar to the original steady-state levels after 2 hrs (88±8%). In contrast, following knockdown of SNAP-23, the rate of KCa2.3 trafficking to the PM was significantly slowed, i.e., KCa2.3 was only 49±3% of the control, steady-state levels (P<0.05). This result indicates that SNAP-23 is also required for the proper trafficking of KCa2.3 in to the PM along the biosynthetic pathway.

## Discussion

KCa2.3 has been shown to play a significant role in a wide array of physiological processes such that understanding not only the regulation and gating of this channel, but also its cell biology is imperative to fully appreciating its role in the cells and tissues where it is expressed. As is clear, during agonist stimulation, both the open probability (P_o_) of a channel, as well as the number of channels (N) in the membrane, is deterministic in any physiological response. Based on this tenet, we and others have undertaken studies to elucidate the mechanisms by which the number of KCa2.3 channels at the PM are regulated. The first clue to the mechanism by which KCa2.3 is retrieved from the PM came from the studies of Absi et al. [24], in which they demonstrated KCa2.3 is localized to caveolin-rich domains in vascular endothelial cells, using IF and co-IP studies. We subsequently demonstrated KCa2.3 is rapidly endocytosed from the PM and enters a recycling compartment from which the channel is returned to the PM with a time constant of ~5 min [25]. We further showed the recycling of KCa2.3 was dependent upon Rab35 as well as the Rab-GAP, EPI64C. Finally, we demonstrated deletion of a small domain within the cytoplasmic N-terminus of KCa2.3 altered the association of the channel with a component of the recycling endosome, RME-1 (EHD1), indicative of a role for this protein in the recycling of KCa2.3 [25]. More recently, we demonstrated the endocytosis of KCa2.3 from the PM depends upon caveolin-1, dynamin II and Rab5 [26]. That is, knockdown and/or overexpression of dominant negative forms of these proteins both increased steady-state PM expression of KCa2.3 and also directly inhibited KCa2.3 endocytosis [26]. Further, Lin et al. [27] demonstrated methyl-β-cyclodextrin-dependent depletion of membrane cholesterol inhibited KCa2.3 endocytosis and recycling, consistent with caveolar localization. These authors also demonstrated this process was regulated by Ca^2+^, such that increasing Ca^2+^ inhibited the endocytosis of KCa2.3.

The studies above have provided mechanistic insight in to the initial steps by which KCa2.3 is endocytosed as well as the early endosomes and recycling endosomes it traverses as it returns to the PM. However, this leaves open the question of what are the SNARE proteins involved in the re-insertion of KCa2.3 in to the PM? The PM SNAREs, Syntaxin-4 and SNAP-23 have previously been shown to reside in caveolin-rich domains in endothelial cells [33]. Given that KCa2.3 resides in these caveolin-rich domains prior to endocytosis we speculated that, following recycling, these SNARE proteins would play a role in KCa2.3 re-insertion back in to this domain. Initially, we demonstrated, using both membrane footprints as well as co-IP studies (Fig 1), KCa2.3 is closely associated with both Syntaxin-4 and SNAP-23 in caveolin-1-rich domains in the PM, indicative of these proteins minimally being in the same membrane compartment. However, these experiments cannot address whether KCa2.3 physically associates with Syntaxin-4 and SNAP-23. Many SNAREs have been shown to physically interact with a variety of ion channels. This includes other K^+^ channels such as the K_ATP_ [35], Kv2.1 [36] and ROMK [37] channels. These interactions have been shown to influence channel insertion in to the PM [38] as well as alter the gating properties of the channel [35,36]. Interestingly, recent studies have shown that disease linked mutations in channel domains that bind SNAREs alter how SNAREs regulate channel function [39,40]. While we show here that SNAP-23 plays an important role in PM insertion, it is not clear from our data whether a reduction in SNAP-23 expression has a potentially additional effect on KCa2.3 channel activity. Further studies are required to determine whether a domain within KCa2.3 directly interacts with these SNARE proteins to facilitate channel insertion in to the PM and whether these interactions persist to influence KCa2.3 channel gating at the PM. Nevertheless, the current study confirms a functional relationship between SNARE expression and KCa2.3 expression and function. That is, we demonstrate that overexpression of Syntaxin-4 and SNAP-23 significantly increase KCa2.3 expression in the PM (Fig 2), whereas knockdown of these SNAREs decreases expression of PM KCa2.3, as assessed both biochemically and electrophysiologically (Figs 3–5).

A decrease in steady-state KCa2.3 expression in the PM could be caused by a decrease in the rate of insertion of channels trafficking from the Golgi to the PM with no change in the retrieval and eventual degradation of channels from the PM. Alternately, we previously demonstrated that ~25% of PM localized KCa2.3 is endocytosed in 5–10 min and recycled back to the PM, such that a decrease in the recycling rate would result in a net decrease in steady-state PM KCa2.3. We demonstrate shRNA-mediated knockdown of SNAP-23 completely eliminates KCa2.3 recycling (Fig 6) while also slowing the Golgi-to-PM delivery of KCa2.3 (Fig 7). We speculate the decrease in steady-state KCa2.3 expression observed following knockdown of SNAP-23 (Fig 4) results from both decreased PM delivery as well as recycling, such that the channel is targeted for degradation. Indeed, we previously demonstrated expression of dominant negative RME-1, which abrogates KCa2.3 recycling, reduces total PM expression of KCa2.3 by 30% [25], consistent with this hypothesis. These results suggest membrane fusion of vesicles containing KCa2.3, whether from recycling endosomes or Golgi derived vesicles require the same SNARE machinery. Interestingly, proteins destined for the PM along the biosynthetic route, in both polarized and non-polarized cells, can traffic either directly to the PM or via recycling endosomes [41–43]. We previously demonstrated the closely related KCa3.1 channel does not recycle at the PM [25,29,30] and it does not traverse through transferrin receptor- or RME-1-positive recycling endosomes on the way to the basolateral membrane in polarized epithelia [28]. It is not known whether KCa2.3 traffics directly to the PM following Golgi exit or if it is targeted to the Rab35/RME-1-positive recycling endosomes prior to being inserted in to caveolin-rich regions of the PM. Given that SNAP-23 knockdown inhibits both trafficking itineraries, we speculate that KCa2.3 is targeted to recycling endosomes after which it is trafficked to the PM, although further experiments will be required to directly assess this possibility.

**Fig 8:**
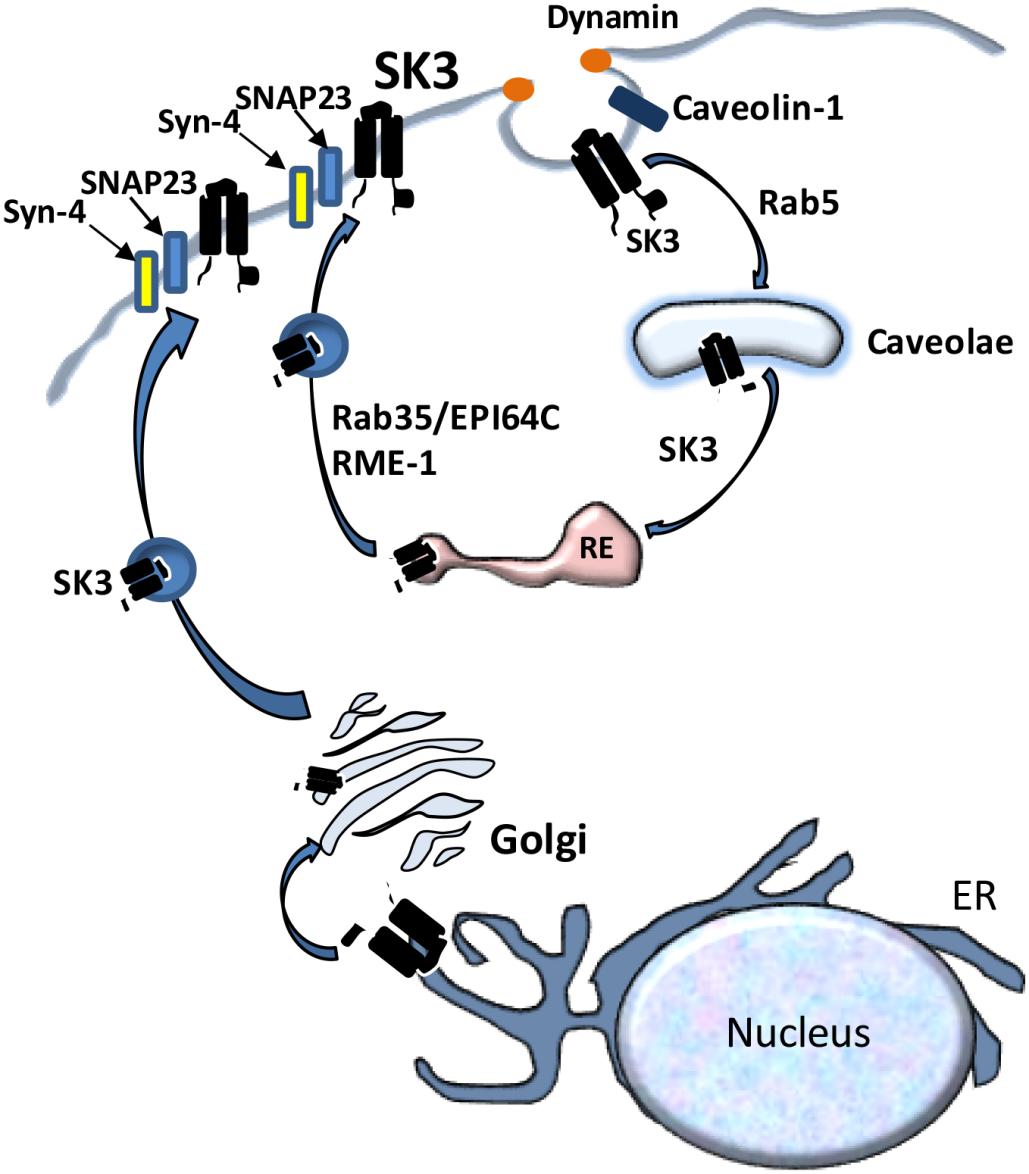
Schematic model of the proposed endocytosis and recycling of KCa2.3 at the plasma membrane. See text for details.

Based on data in the current manuscript, together with previously published data, we propose the model shown in Fig 8 for the recycling of KCa2.3 at the PM. KCa2.3 resides in the PM in caveolin-1- and cholesterol-rich domains [24,26,27]. The endocytosis of KCa2.3 requires caveolin-1, dynamin II and Rab5, after which KCa2.3 is localized to caveolae and Rab-5-positive early endosomes [26]. Subsequently, KCa2.3 enters Rab35/EPI64C- and RME-1-containing recycling endosomes [25]. Herein, we demonstrate the fusion of these KCa2.3-containing recycling endosomes with the PM requires Syntaxin-4 and SNAP-23; completing the recycling loop. It is also known that other proteins required for KCa2.3 function in endothelia, including TRPV4 and Connexin-43 (CX43) are localized to caveolae [44,45]. Whether these proteins traffic together as a macro-molecular complex from the PM is not known. However, CX43 has been shown to have a short membrane half-life in several cell types [46,47] compared to the long half-life we reported for KCa2.3 [25]. Also, TRPV4 has been shown to reside in intracellular vesicles that traffic in to the PM in response to increasing intracellular Ca^2+^ [48]; nearly doubling total PM expression of this Ca^2+^ channel. Interestingly, we found that increasing intracellular Ca^2+^ results in a doubling of the amount of TRPV4 associated specifically with PM localized KCa2.3, as assessed by co-IP, suggesting the trafficking of these proteins may be linked during a rise in Ca^2+^ (CAB and DCD, unpublished observations). Thus, while it is possible these proteins are initially endocytosed together from caveolin-1-rich PM microdomains in to caveolae, it is likely they would then be sorted in to unique vesicle pools and progress along unique intracellular itineraries. While our studies herein close the recycling loop for KCa2.3, additional studies are required to determine how KCa2.3 is successfully targeted in to the macromolecular complex that defines the functional unit required for a physiological response.
